# Body mass index and health-related quality of life among young Swiss men

**DOI:** 10.1186/1471-2458-13-1028

**Published:** 2013-10-30

**Authors:** Michelle Dey, Gerhard Gmel, Meichun Mohler-Kuo

**Affiliations:** 1 Institute of Social and Preventive Medicine, University of Zurich, Hirschengraben 84, Zurich 8001, Switzerland; 2 Alcohol Treatment Centre, Lausanne University Hospital CHUV, Av. Beaumont 21 bis, Pavillon 2, Lausanne 1011, Switzerland

**Keywords:** Health-related quality of life, Body mass index, Physical activity, Alcohol, Cigarettes, Cannabis

## Abstract

**Background:**

Studies about the association between body mass index (BMI) and health-related quality of life (HRQOL) are often limited, because they 1) did not include a broad range of health-risk behaviors as covariates; 2) relied on clinical samples, which might lead to biased results; and 3) did not incorporate underweight individuals. Hence, this study aims to examine associations between BMI (from being underweight through obesity) and HRQOL in a population-based sample, while considering multiple health-risk behaviors (low physical activity, risky alcohol consumption, daily cigarette smoking, frequent cannabis use) as well as socio-demographic characteristics.

**Methods:**

A total of 5 387 young Swiss men (mean age = 19.99; standard deviation = 1.24) of a cross-sectional population-based study were included. BMI was calculated (kg/m^2^) based on self-reported height and weight and divided into ‘underweight’ (<18.5), ‘normal weight’ (18.5-24.9), ‘overweight’ (25.0-29.9) and ‘obese’ (≥30.0). Mental and physical HRQOL was assessed via the SF-12v2. Self-reported information on physical activity, substance use (alcohol, cigarettes, and cannabis) and socio-demographic characteristics also was collected. Logistic regression analyses were conducted to study the associations between BMI categories and below average mental or physical HRQOL. Substance use variables and socio-demographic variables were used as covariates.

**Results:**

Altogether, 76.3% were normal weight, whereas 3.3% were underweight, 16.5% overweight and 3.9% obese. Being overweight or obese was associated with reduced physical HRQOL (adjusted OR [95% CI] = 1.58 [1.18-2.13] and 2.45 [1.57-3.83], respectively), whereas being underweight predicted reduced mental HRQOL (adjusted OR [95% CI] = 1.49 [1.08-2.05]). Surprisingly, obesity decreased the likelihood of experiencing below average mental HRQOL (adjusted OR [95% CI] = 0.66 [0.46-0.94]). Besides BMI, expressed as a categorical variable, all health-risk behaviors and socio-demographic variables were associated with reduced physical and/or mental HRQOL.

**Conclusions:**

Deviations from normal weight are, even after controlling for important health-risk behaviors and socio-demographic characteristics, associated with compromised physical or mental HRQOL among young men. Hence, preventive programs should aim to preserve or re-establish normal weight. The self-appraised positive mental well-being of obese men noted here, which possibly reflects a response shift, might complicate such efforts.

## Background

Excessive weight, and especially obesity, is a major public health concern for several reasons. First, the number of individuals who are either overweight or obese has reached epidemic proportions in the United States [1] and many European countries [2]. Relative to other industrialized countries, Switzerland’s prevalence of excess weight is relatively low [2], but nevertheless has increased over the years [3]. In a recent Swiss Health Survey (SHS 2007), 30.4% of the total adult population were overweight and an additional 8.5% obese [4]. Second, excessive weight is associated with increased morbidity and mortality [5].

However, deviations from normal weight might not only affect the physical health of a person, but also more psychosocial domains. In order to comprehensively understand a person’s subjective perspective on all these multiple life domains, the concept of *health-related quality of life* (HRQOL) can be used. Published studies that have utilized this outcome indicate that being overweight or at least being obese (mostly assessed via body mass index; BMI) is related to compromised HRQOL [6-37].

In addition to BMI and waist circumference, *health-risk behaviors* contribute further to the prediction of reduced HRQOL. This has been demonstrated repeatedly for low physical activity [10,12,13,15,19-21,33,35]. To date, other health-risk behaviors, such as substance use, have infrequently been (thoroughly) considered as covariates. Furthermore, it is evident that investigators who included such health-risk behaviors primarily focused on smoking cigarettes [6,10,12-17,19,22,26,36,38], whereas other substances like alcohol were only rarely incorporated [6,22]. Concurrently including multiple health-risk behaviors (i.e., not only physical inactivity but also substance use), while studying the associations between BMI categories and HRQOL, might be especially important among young men, because risky substance use is quite common in this age-sex-group [39]. Furthermore, the just-mentioned health-risk behaviors seem to be associated with BMI (e.g., [10,19,40-45]), as well as with negative HRQOL (e.g., [10,12,13,15,19-21,33,35,39],[46-49]).

Besides not including a broad range of health-risk behaviors, existing studies about the association between BMI and HRQOL are limited, because they often relied upon clinical samples, which might have led to an overestimation of the negative effect of excessive weight on HRQOL [37,50]. Furthermore, underweight individuals were often neglected, even though they also suffer from compromised HRQOL [9-11,14-16,19,26,28,30,31,35],[36,38], an effect that seems to be especially pronounced among men [10,15].

Due to the above-mentioned limitations, the main aim of the present analysis was to examine associations between BMI (from underweight through obesity) and HRQOL, while thoroughly considering multiple health-risk behaviors (low physical activity, risky alcohol consumption, daily cigarette smoking, frequent cannabis use) in a population-based sample of young Swiss men.

## Methods

### Study design

For this study, we drew data from the *‘Cohort Study on Substance Use Risk Factors’ (C-SURF)*, the study protocol of which was approved by the Ethics Committee for Clinical Research at Lausanne University Medical School (protocol number 15/07). The study participants were recruited at three of a total of six centers that recruit men for military service, representing 21 of 26 Swiss cantons. Virtually all Swiss men must go through this recruitment process to determine their eligibility for military, civil or no service at roughly the age of 19 years (i.e., no pre-selection to army conscription exists). Army centers were only used to enroll participants into the study. Hence, the study is independent of the army. Of the 13 245 conscripts who were seen by the research staff, 57.1 % gave informed consent. A questionnaire was sent to these 7 563 men approximately two weeks after the army recruitment process. Of this number, 5 990 (response rate = 79.2 %) subsequently filled out the questionnaire between September 2010 and March 2012.

### Measurements

All variables were based on the self-reports of participants.

### Socio-demographics

*Age* (‘younger than 20 years’ vs. ’20 years or older’) and *type of residence* (‘rural’ (< 10 000 inhabitants) vs. ‘urban’ (≥ 10 000 inhabitants)) were used. Participants also were asked about the *financial situation of their family* relative to other families living in Switzerland. The original answer format (1 ‘very significantly above average’ - 7 ‘very significantly below average’) was re-coded into the following three categories: *‘above average’* (former categories 1 to 3), *‘average’* (former category 4) and *‘below average’* (former categories 5 to 7).

### BMI

Self-reports about height and weight were used to calculate BMI (kg/m^2^), which was then divided according to the World Health Organization’s [51] protocol into ‘underweight’ (<18.5), ‘normal weight’ (18.5-24.9), ‘overweight’ (25.0-29.9) and ‘obese’ (≥30.0).

### Health-risk behaviors

#### Low physical activity

Physical activity was assessed using the *‘International Physical Activity Questionnaire – short form’*[52], divided into low, moderate and high (based upon the particular physical activity that a given person performed during the last week and the average time spent performing this/these activity/activities). People who reported physical activity of more than 16 hours per day were defined as outliners and were excluded from further analysis [53]. For the purposes of the present article, the three activity categories were dichotomized into *‘moderate to high physical activity’* (coded as 0) vs. *‘low physical activity’* (coded as 1).

#### Alcohol: risky single-occasion drinking (RSOD)

Men were, according to the frequency and amount of their alcohol intake, classified as *‘not at-risk RSOD’* (which also includes those who consume no alcohol at all; coded as 0) vs. *‘at-risk RSOD’* (coded as 1). *At-risk RSOD* was defined as consuming at least 6 standard drinks on a single occasion at least monthly. Pictures of standard drinks containing 10–12 grams of pure alcohol were provided for reference.

#### Daily cigarette smoking

Cigarette smoking was dichotomized into *‘non- or occasional smoking’* (coded as 0) vs. *‘daily smoking’* (coded as 1).

#### At-risk cannabis use

Cannabis use was categorized into *‘not at-risk cannabis use’* (including those who do not use cannabis or use it no more than once per week; coded as 0) vs. *‘at-risk cannabis use’* (using cannabis more than once per week; coded as 1).

### HRQOL

HRQOL was assessed using the *‘Medical Outcomes Study 12-Item Short Form Survey Instrument (SF-12v2)’*[54]. Both the calculated ‘physical component summary’ (PCS) and the ‘mental component summary’ (MCS) were linearly transformed into norm-based scores (mean = 50; SD = 10) and subsequently – due to their non-normal distribution – dichotomized into *‘(above) average HRQOL’* (greater than or equal to 45; coded as 0) and *below average HRQOL* (less than 45; coded as 1). These cut-offs were based upon assigning ½ a standard deviation as a clinically meaningful difference in HRQOL [55].

### Statistical analysis

Altogether, 603 of the 5 990 participating men were excluded from analysis, due to missing data (e.g., regarding their height or weight) or being outliers in the assessment of physical activity (see above). Consequently, the final analytical sample consisted of 5 387 men. Socio-demographic characteristics of German- vs. French-speaking conscripts were compared via chi-square analysis (categorical variables) and t-tests (continuous variables). Chi-square analysis was used to examine for associations between socio-demographic variables and health-risk behaviors on one hand and BMI categories on the other hand. As a final step, logistic regression analyses were conducted to assess for associations between the here-used predictors (BMI as a categorical value, health-risk behaviors, and socio-demographic variables) and HRQOL (PCS and MCS). Both crude and adjusted odds ratios (OR) were calculated.

## Results

### Participants

Of the total 5 387 men analyzed, 2 400 were from the German-speaking and 2 987 from the French-speaking region of Switzerland. Compared to German-speaking conscripts (mean age = 19.65, SD = 1.08), French-speaking men tended to be older (mean age = 20.27, SD = 1.29; *t*_*5375.9*_ = −19.25; *p* < .001). German- and French-speaking men also differed in the self-reported financial situation of their families (*Χ*^*2*^_*2*_ = 254.01; p < .001), with German-speaking men more often estimating their family’s financial situation as being above average (54.9 % vs. 35.9 %), but less often in the average range (29.7 % vs. 50.7 %). The percentage of those describing their financial situation as being below average did not differ to any great extent by linguistic region (German-speaking: 15.5%; French-speaking: 13.4 %).

### Socio-demographics/health-risk behaviors and BMI

The associations between socio-demographics and health-risk behaviors on the one hand and BMI on the other hand are depicted in Table 1. Altogether, 76.3 % of the sample was of normal weight, while 3.3 % of participants were underweight, 16.5 % overweight and 3.9 % obese. Although young men from both the German- and French-speaking regions of Switzerland had a similar percentage of normal weight individuals (76.3 %), the percentage with overweight was higher in the German-speaking region, whereas the prevalence rates for being underweight and obese were higher in the French. Relative to men 20 years old or older, younger men more often had a normal weight and were less often overweight or obese. The percentage of underweight did not vary by age. The prevalence of normal weight increased with increasing financial means of one’s family, while the percentage of overweight and obesity decreased. Furthermore, being underweight was more common when the financial situation of the family was described as being below average, relative to an average or above average financial situation.

**Table 1:** Association between socio-demographics/health-risk behaviors and BMI

		**BMI categories**					
	***n***	***Underweight (%)***	***Normal weight (%)***	***Overweight (%)***	***Obese (%)***	**X**^**2**^	**p**
	***Total = 5 387***	***3.3***	***76.3***	***16.5***	***3.9***		
*Linguistic region*							
German-speaking	*2 400*	2.3	76.3	17.8	3.5	18.26	<.001
French-speaking	*2 987*	4.1	76.3	15.5	4.1		
*Age*							
< 20 years	*3 209*	3.4	78.5	14.6	3.5	25.46	<.001
≥ 20 years	*2 178*	3.2	73.1	19.3	4.4		
*Family financial situation*							
Above average	*2 390*	3.1	77.8	15.9	3.1	17.09	.009
Average	*2 226*	3.2	76.4	16.2	4.2		
Below average	*771*	4.3	71.3	19.2	5.2		
*Physical activity*							
Moderate/high	*4 906*	3.2	76.7	16.5	3.6	16.00	.001
Low	*481*	4.6	72.1	16.4	6.9		
*At-risk RSOD*							
No	*2 930*	4.1	75.2	16.4	4.3	16.43	.001
Yes	*2 457*	2.4	77.7	16.6	3.3		
*Daily cigarette smoking*							
No	*4 295*	3.3	77.3	16.1	3.3	24.89	<.001
Yes	*1 092*	3.3	72.3	18.1	6.2		
*At-risk cannabis use*							
No	*4 890*	3.2	75.9	16.9	4.0	10.20	.017
Yes	*497*	4.4	80.3	12.7	2.6		

*Note*: BMI, Body mass index; RSOD, Risky single-occasion drinking.

Significant associations also were identified between all health-risk behaviors (i.e., low physical activity; at-risk RSOD; daily cigarette smoking; at-risk cannabis use) and BMI categories (Table 1). Men with a low level of physical activity were less often of normal weight but more often underweight or obese than men who were physically more active. The percentage overweight did not differ between men with a low versus moderate to high physical activity level. Men who were at-risk RSOD were less often underweight or obese but more often normal weight than men not reporting this risky drinking pattern. No differences in overweight rates were observable between at-risk versus not at-risk RSOD. Daily cigarette smokers were more often overweight or obese, but less often normal weight than non- or occasional smokers. Lastly, at-risk cannabis users were, relative to not at-risk users, more often underweight or normal weight, but less often overweight or obese.

### HRQOL

Crude ORs for all HRQOL predictors (BMI, socio-demographic characteristics, and health-risk behaviors) are described in Table 2. Adjusted ORs for the multiple logistic regression analyses (adjusted for all predictors) are also reported. The crude and adjusted ORs always point in the same direction. However, the adjusted ORs mostly were attenuated.

**Table 2:** Logistic regression models for HRQOL versus BMI

	**Below average physical component summary (PCS)**^**a**^			**Below average mental component summary (MCS)**^**a**^		
	***n *** **(%)**	**Crude OR [CI]**	**Adjusted OR**^**b **^**[CI]**	***n *** **(%)**	**Crude OR [CI]**	**Adjusted OR**^**b **^**[CI]**
**Total (*n *** **=5 387)**	**299 (5.6)**			**1 404 (26.1)**		
*BMI*						
Normal weight	192 (4.7)	1.00	1.00	1 061 (25.8)	1.00	1.00
Underweight	15 (8.4)	1.87 [1.08-3.23]^*^	1.59 [0.91-2.78]	65 (36.3)	1.64 [1.20-2.24]^**^	1.49 [1.08-2.05]^*^
Overweight	65 (7.3)	1.61 [1.20-2.15]^***^	1.58 [1.18-2.13]^**^	236 (26.5)	1.04 [0.88-1.23]	1.05 [0.88-1.24]
Obese	27 (13.0)	3.05 [1.98-4.68]^***^	2.45 [1.57-3.83]^***^	42 (20.2)	0.73 [0.52-1.03]	0.66 [0.46-0.94]^*^
*Linguistic region*						
German-speaking	102 (4.3)	1.00	1.00	425 (17.7)	1.00	1.00
French-speaking	197 (6.6)	1.59 [1.25-2.03]^***^	1.42 [1.10-1.84]^**^	979 (32.8)	2.27 [1.99-2.58]^***^	2.07 [1.80-2.37]^***^
*Age*						
< 20 years	157 (4.9)	1.00	1.00	702 (21.9)	1.00	1.00
≥ 20 years	142 (6.5)	1.36 [1.07-1.71]^*^	1.10 [0.86-1.41]	702 (32.2)	1.70 [1.50-1.92]^***^	1.43 [1.26-1.63]^***^
*Family financial situation*						
Above average	102 (4.3)	1.00	1.00	538 (22.5)	1.00	1.00
Average	140 (6.3)	1.51 [1.16-1.96]^**^	1.29 [0.98-1.69]	618 (27.8)	1.32 [1.16-1.51]^***^	1.10 [0.95-1.26]
Below average	57 (7.4)	1.79 [1.28-2.50]^***^	1.47 [1.04-2.08]^*^	248 (32.2)	1.63 [1.37-1.95]^***^	1.48 [1.23-1.78]^***^
*Physical activity*						
Moderate/high	231 (4.7)	1.00	1.00	1 248 (25.4)	1.00	1.00
Low	68 (14.1)	3.33 [2.50-4.45]^***^	2.99 [2.22-4.01]^***^	156 (32.4)	1.41 [1.15-1.72]^***^	1.40 [1.14-1.73]^**^
*At-risk RSOD*						
No	190 (6.5)	1.00	1.00	731 (24.9)	1.00	1.00
Yes	109 (4.4)	0.67 [0.53-0.85]^***^	0.70 [0.54-0.90]^**^	673 (27.4)	1.14 [1.01-1.28]^*^	1.14 [1.00-1.30]^*^
*Daily cigarette smoking*						
No	215 (5.0)	1.00	1.00	1 065 (24.8)	1.00	1.00
Yes	84 (7.7)	1.58 [1.22-2.05]^***^	1.43 [1.07-1.91]^*^	339 (31.0)	1.37 [1.18-1.58]^***^	1.06 [0.90-1.25]
*At-risk cannabis use*						
No	263 (5.4)	1.00	1.00	1 202 (24.6)	1.00	1.00
Yes	36 (7.2)	1.37 [0.96-1.97]	1.29 [0.87-1.93]	202 (40.6)	2.10 [1.74-2.54]^***^	1.85 [1.50-2.29]^***^

*Note*: BMI, Body mass index; CI, 95 % Confidence interval; OR, Odds ratio; RSOD, Risky single-occasion drinking; ^*^ = p ≤ .05; ^**^ = p ≤ .01; ^***^ = p ≤ .001.

^a^refers to the SF-12v2 summary scores; ^b^ model includes all predictors (BMI categories, socio-demographic characteristics, health-risk behaviors).

Deviations from normal weight predicted compromised HRQOL, even after adjusting for covariates. Being overweight and being obese were associated with a reduced physical score in terms of HRQOL (PCS), whereas those who were underweight were more likely to have a below average mental score (MCS). Notably, relative to normal weight men, obese men were less likely to report a compromised MCS.

In addition to BMI, socio-demographic characteristics were associated with HRQOL. In the adjusted models, French-speaking conscripts were more likely to report a compromised PCS and MCS than German-speaking conscripts. Furthermore, conscripts who were 20 years or older were, relative to younger men, more likely to report a compromised MCS. Lastly, more men who described the financial situation of their family as being below average claimed a reduced PCS and MCS than those from families whose financial situation was above average.

In addition, all health-risk behaviors were related to HRQOL. In the adjusted models, a low physical activity level significantly contributed to the prediction of compromised PCS and – to a smaller extent – MCS. In addition, at-risk RSOD was associated with a higher likelihood of experiencing reduced MCS. However, men with at-risk RSOD were less likely to report compromised PCS. Lastly, daily cigarette smoking was linked to poorer PCS, whereas at-risk cannabis use negatively affected MCS.

## Discussion

In the present population-based study, 76.3% of young Swiss men were of normal weight, whereas 3.3% were underweight, 16.5% overweight and an additional 3.9% obese, percentages that are similar to those identified in the 2007 Swiss Health Survey [4] among males in the same age range (18 to 31 years: underweight: 2.1%; normal weight: 69.8%; overweight: 24.2%; obese: 3.9%). In terms of how weight affects HRQOL, we found that deviations from normal weight in both directions were associated with compromised HRQOL. Both overweight and obese men claimed reduced physical HRQOL relative to normal weight individuals, whereas underweight conscripts suffered from compromised mental HRQOL. Surprisingly, obese men reported better mental HRQOL. Health-risk behaviors as well as socio-demographic characteristics contributed to the prediction of HRQOL, as well.

### BMI and HRQOL

As described above, being overweight or obese was associated with *compromised physical HRQOL* (with small and medium effect sizes, respectively; [56]). This finding is compatible with other studies that indicate that excess weight is solely or at least to a larger extent related to reduced physical than to reduced mental HRQOL [9,10,16-19,21,22,24-26,29-31,33],[35,37]. However, our finding that obese men have *better mental HRQOL* than men of normal weight (even though this effect was relatively small; [56]) contradicts most earlier studies that have demonstrated either a negative association or no association at all between obesity and mental HRQOL [7,8,10,12,18-21,23-32,34,35],[37,38] or related HRQOL domains (e.g., self-esteem; [11]). Only a few investigations have revealed that (some) obese people have slightly better mental HRQOL than those of normal weight [16,17,22]. Various explanations for these diverging results related to obesity and positive versus negative mental HRQOL must be considered:

1) *Sampling strategies*: While our study and others that have identified a positive association between obesity and mental HRQOL [16,17,22] analyzed population-based samples, at least some of the studies that found no or a negative association used clinical samples [7,8,18,25,27,32,34], which seem to be characterized by more pronounced mental health and related problems [37,50].

2) *Gender*: The positive association we discovered between obesity and enhanced mental HRQOL also might have occurred because we only included men in our sample. This hypothesis is based upon observations that i) the negative effect of obesity on mental HRQOL sometimes has been limited to females [6,21,23,28] and; ii) the positive effect of obesity on mental HRQOL was only discovered among men in the study by Lopez-Garcia et al. [22].

3) *Age*: Our sample consisted of young adults, and it has been demonstrated previously that this age group seems not to suffer from reduced mental HRQOL [21].

4) *Degree of obesity:* The described finding also might have been because only a small number of obese individuals in the current sample had extremely high BMI scores (results not shown), and it is possible that only individuals who are dramatically obese suffer from obesity-related reduced mental HRQOL [19,25,26,29]. Additional analysis fails to support this hypothesis, however, since the percentage of obese men who experienced compromised mental HRQOL decreased with increasing BMI.

5) *Response shift:* One final explanation for this somewhat unexpected association between obesity and more positive mental HRQOL is the so-called *response shift*[57]. That is, comparable to people suffering from a severe physical illness, obese men may have adapted to their condition and, thereby, come to positively appraise their mental HRQOL. The likelihood that such a response shift occurs probably increases with increasing BMI. This assumption is not only supported by the above-described finding that the mental HRQOL increased with increasing levels of obesity, but also by the result that overweight men reported a slightly lower mental HRQOL than normal weight men.

On the flip side, the association we demonstrated between low weight and below average mental HRQOL (small effect size; [56]) was in line with previous findings [9,19,26,28,31], at least in a male subsample [16]. Furthermore, Friedlander et al. has described compromised HRQOL in a related domain, namely self-esteem [11]. Our result might, among other things, have occurred because being underweight as a man is incompatible with the usually-aspired male ideal of being muscular [58]. That being underweight was not associated with compromised physical HRQOL in the adjusted model suggests that – in most of our underweight men – no severe physical health condition was present that might have caused either their low weight or any pronounced reduction in their mental HRQOL.

### Health-risk behaviors

Consistent with previous studies, we found that low levels of *physical activity* are associated with a less favorable BMI (i.e., lower prevalence of normal weight; [10,19]). And with worse HRQOL [10,12,13,15,19-21,33,35,46].

Regarding *alcohol*, various studies indicate that drinking large quantities on a single occasion is associated with an increased BMI [40,41]. Our results differ from this pattern, possibly because men with a risky drinking pattern were physically more active than men who did not report at-risk RSOD (results not shown; comparable to [39]). In other words, it is possible that men with a risky drinking pattern did not gain weight because they participated in moderate to high-level physical activity. That a risky drinking pattern only is associated with compromised HRQOL in the mental but not physical domain is consistent with the results of other studies [48,49].

*Daily smokers* were, in our sample, more often overweight or obese than non- or occasional smokers, a result that also has been observed in other studies that included young adults (e.g., [45]). The reduced physical HRQOL among smokers also has been reported previously [12,19], one potential explanation for it being the negative effect this health-risk behavior has on respiratory function. Initially, the crude OR indicated that daily cigarette smoking was associated with reduced mental HRQOL. However, the association became non-significant after we adjusted for other covariates. This result contradicts earlier findings [12,19,46,49]. However, the non-significant result was primarily due to adjusting for other covariates like the risky use of cannabis.

Lastly, that *at-risk cannabis users* both had a lower prevalence of excessive weight and reduced mental well-being is consistent with earlier studies (see [43,44] and [47]).

### Socio-demographic characteristics

In our sample of 5 387 young Swiss men, socio-demographic variables were found to be associated with both BMI and HRQOL. The effect of *linguistic region* may have been due to cultural differences. It is, for instance, possible that people from the French-speaking region more often chose extreme answers, which would explain their higher prevalence of low weight and obesity, as well as a higher percentage claiming below average HRQOL. Furthermore, younger men overall had a more favorable BMI distribution and better mental HRQOL than men 20 years or older. One possible explanation for this latter finding relates to situational changes (e.g., leaving home; educational/vocational changes) that tend to occur at about age 20, which might make it more difficult for someone to remain as physically active (e.g., due to less leisure time). This reduction in physical activity and/or leisure time, in turn, might induce stress that compromises mental HRQOL. Lastly, the negative impact of low socio-economic status (assessed in term of the financial situation of the participant’s family) on BMI, as well as on compromised physical and mental HRQOL, is consistent with earlier reports (e.g., [1,12,13,19]).

### Limitations

The following limitations of the present study must be considered: First, only men were assessed, even though earlier investigations have demonstrated differing associations between BMI and HRQOL for both men and women (e.g., [6,10,17,21,28]). Second, no data were available about physical health problems that are known to be associated with increased BMI. Hence, the demonstrated associations between BMI and HRQOL may have been affected by certain weight-related co-morbidities (e.g., [6,8,9,38]). Additionally, no data were available about other potentially-influential variables, such as *perceived weight status* (i.e., how a person judges his/her own weight), which may be more strongly associated with HRQOL than BMI [14]. Third, all data were self-reported, and self-reports may be biased. For instance, subjects may have overestimated their height [59], which subsequently would have led to an underestimation of their BMI. However, as demonstrated by Herman et al. [15], the prevalence of compromised HRQOL is similar regardless of whether BMI is measured or self-reported. Fourth, BMI fails to distinguish between lean and fat body mass [60]. Hence, it cannot be ruled out that, in some men, a BMI in the overweight range reflected increased muscle mass due to high levels of physical activity [61]. Lastly, causality cannot be inferred due to the cross-sectional nature of data collection. For example, though it is possible that deviations from normal weight lead to compromised HRQOL, it also is possible that compromised HRQOL leads to deviations from normal weight.

## Conclusions

These limitations notwithstanding, among young men living in Switzerland, it appears that being overweight or obese is associated with compromised physical HRQOL, while being underweight is associated with reduced mental HRQOL. These findings hold true even after controlling for important health-risk behaviors and socio-demographic characteristics. Hence, preventive programs should aim not just to promote weight loss in those who are overweight, but weight gain in those whose weights are below normal. This being said, it also must be kept in mind that obese young men might tend to appraise their mental HRQOL positively, an effect that could be explained by response shift. Such a change in the appraisal of one’s HRQOL might reduce an obese man’s willingness to lose weight.

From a practical/interventional standpoint, the present study provided insights that may be used for the conceptualization of public health programs. For example, promoting a moderate to high physical activity level could be a potent starting point for such a program, because this health-promoting behavior not only positively affects BMI, it also enhances physical and mental HROQL. Furthermore, we found that excessive weight and frequent smoking often co-occur among young men; hence, both these major health-risk factors must be targeted. Lastly, programs targeting weight normalization should especially focus upon people of low socio-economic status, because they are characterized by a worse distribution of BMI and particularly compromised HRQOL.

With respect to future research, the present investigation demonstrates how important it is to include multiple health-risk behaviors when studying the relationship between BMI and HRQOL. Especially when studying young men, such associations must be taken into account.

## Abbreviations

BMI: Body mass index; HRQOL: Health-related quality of life; MCS: Mental component summary; PCS: Physical component summary; OR: Odds ratios; RSOD: Risky single-occasion drinking; SHS: Swiss Health Survey.

## Competing interests

The authors declare that they have no competing interests.

## Authors’ contributions

MD conceptualized the manuscript, performed statistical analyses, and drafted the manuscript. GG is the principal, and MMK co-principal investigator of C-SURF and both authors helped to conceptualize and draft the manuscript. All authors read and approved the final manuscript.

## Pre-publication history

The pre-publication history for this paper can be accessed here:

http://www.biomedcentral.com/1471-2458/13/1028/prepub
